# Awake and Sleep Bruxism Prevalence and Their Associated Psychological Factors in First-Year University Students: A Pre-Mid-Post COVID-19 Pandemic Comparison

**DOI:** 10.3390/ijerph20032452

**Published:** 2023-01-30

**Authors:** Álvaro Edgardo Osses-Anguita, Teresa Sánchez-Sánchez, Xabier A. Soto-Goñi, María García-González, Francisco Alén Fariñas, Rosana Cid-Verdejo, Eleuterio A. Sánchez Romero, Laura Jiménez-Ortega

**Affiliations:** 1 Department of Conservative and Prosthetic Dentistry, Faculty of Odontology, Complutense University of Madrid, 28040 Madrid, Spain; 2 Facultad de Odontología y Ciencias de la Rehabilitación, Universidad San Sebastián, Lientur 1457, Concepción 4080871, Chile; 3 Department of Psychobiology and Behavioral Sciences Methods, Faculty of Odontology, Complutense University of Madrid, 28040 Madrid, Spain; 4 Departament of Clinical Dentistry, Faculty of Biomedical Sciences, University Europea of Madrid, 28670 Madrid, Spain; 5 Department of Physiotherapy, Faculty of Sport Sciences, Universidad Europea de Madrid, 28670 Villaviciosa de Odón, Spain; 6 Physiotherapy and Orofacial Pain Working Group, Sociedad Española de Disfunción Craneomandibular y Dolor Orofacial (SEDCYDO), 28009 Madrid, Spain; 7 Musculoskeletal Pain and Motor Control Research Group, Faculty of Sport Sciences, Universidad Europea de Madrid, 28670 Madrid, Spain; 8 Department of Physiotherapy, Faculty of Health Sciences, Universidad Europea de Canarias, 38300 Santa Cruz de Tenerife, Spain; 9 Musculoskeletal Pain and Motor Control Research Group, Faculty of Health Sciences, Universidad Europea de Canarias, C/Inocencio García 1, 38300 La Orotava, Spain; 10 Center of Human Evolution and Behavior, UCM-ISCIII, 28029 Madrid, Spain; 11 Psychology and Orofacial Pain Working Group, Sociedad Española de Disfunción Craneomandibular y Dolor Orofacial (SEDCYDO), 28009 Madrid, Spain

**Keywords:** bruxism, stress, anxiety, depression, neuroticism, coping, dental students, COVID-19

## Abstract

There is a broad consensus accepting that psychological variables such as stress, anxiety, or depression play an important role in bruxism. The COVID-19 pandemic has led to an increase in stress, anxiety, and depression levels. The purpose of this study was to evaluate the impact of the COVID-19 pandemic on possible awake and sleep bruxism prevalence and on the psychological factors associated with bruxism, comparing pre-pandemic, pandemic/lockdown, and post-pandemic samples of first-year students. A total of 274 dentistry students from the Complutense University of Madrid participated in the study: 92 from 2018/2019 (pre-pandemic), 90 from 2020/2021 (pandemic), and 92 students from 2021/2022 (post-pandemic) academic years. The participants filled out a thorough battery of validated questionnaires evaluating bruxism and different psychological characteristics, such as anxiety, depression, somatization, personality, and stress coping styles. While sleep bruxism prevalence was significantly higher for the pandemic group, awake bruxism was smaller in comparison to pre-pandemic and post-pandemic groups. The post-pandemic group also presented higher levels of neuroticism and agreeableness personality traits, and positive reappraisal than the pre-pandemic group, with the pandemic group somewhere in between. Additionally, both the pandemic and post-pandemic group showed higher levels of depression and acceptance/resignation coping styles than the pre-pandemic group. Thus, among the three groups of students, the post-pandemic group was the one that showed a larger effect of the pandemic situation in their psychological variables, presenting higher levels of anxiety (state and trait), depression, acceptation/resignation coping style, higher neuroticism (emotional instability trait), and lower agreeableness trait. Nonetheless, the increase of positive reappraisal in the post-pandemic group (an adaptive coping stress style) might be also a sign of recovery. The higher sleep bruxism for the pandemic group might be related to the pandemic situation and lockdown, passively suffered, possibly promoting feelings of impotency, increased levels of depression and acceptance/resignation (normally considered a passive/maladaptive coping style), while acute stressful situations derived from daily personal social interactions might have increased anxiety levels and induced higher levels of awake bruxism observed in both the pre-pandemic and post-pandemic groups. However, further research, including larger and more representative samples, is needed to confirm this possible relationship.

## 1. Introduction

The current international consensus defines bruxism as an activity of the masticatory muscles, being characterized as two different, non-exclusive entities, according to their manifestation in the circadian cycle: awake bruxism, and sleep bruxism. These are phenomena regulated by the central nervous system, of multifactorial origin, with peripheral factors (anatomical or occlusal) playing a secondary role. An expert consensus definition has now been adopted, which provides separate definitions for sleep bruxism and awake bruxism: (1) sleep bruxism is a muscular chewing activity during sleep that is characterized as rhythmic (phasic) or nonrhythmic (tonic) and is not a movement or sleep disorder in otherwise healthy individuals; (2) awake bruxism is a muscular chewing activity during wakefulness that is characterized by repetitive or sustained tooth contact and/or jaw bracketing or thrusting and is not a movement disorder in otherwise healthy individuals [1]. In itself, it is not considered pathological, but it can be a protective or risk factor for other conditions [1,2,3,4]. Its diagnosis is classified according to the level of certainty as possible (self-report) [1,5,6,7,8,9], probable (clinical examination) [1,4,6] and definite (instrumental) [1,4,6]. Anxiety, stress, and depression, among other psychological factors, have been associated with bruxism in various population studies, particularly awake bruxism [5,8,10,11,12,13].

The prevalence of bruxism is as high as 30% of the population [14]. Although it is considered a behaviour rather than a disease (a continuum defined by its frequency, intensity, and duration), in certain circumstances it may be pathological, causing problems such as damaged teeth and orofacial pain, and it is among the possible risk factors in the development of temporomandibular disorders (TMD) [15]. However, a recent systematic review has found that there is insufficient evidence to suggest that patients with temporomandibular joint osteoarthritis are associated with increased sleep disturbances or poorer sleep quality [16].

Additionally, several studies observed biological markers such as increased cortisol, catecholamines, and substance P (SP), associated with psychological alterations [1,5,6]. Furthermore, some studies have shown a causal relationship between stressful activities and bruxism [17]. In this line, it has been shown that participants with muscular TMD exposed to relaxing music decreased their muscular strain while stressful music increased it during spontaneous awake bruxism episodes [18]. In a recent study, it was also found that participants with definitive awake bruxism displayed greater muscular activity when presented with videos and texts with a negative valence, especially when related to pain, than the non-bruxism group [19].

Altogether, it can be hypothesized that stress situations might increase bruxism behavior. Thus, the beginning of university life requires a period of adaptation to a new social context and life demands, eventually being away from family, adjusting to a new group of friends, acquiring new responsibilities, etc. This period can be stressful and generate anxiety [20,21], which might impact the bruxism behavior. Additionally, COVID-19 disease and the consequent confinement due to the sanitary measures taken in Spain and many other countries has resulted in increased levels of anxiety, stress, and depression in the population [22,23,24,25]. Furthermore, recent evidence suggests an intensification of bruxism and TMD symptoms [26], probably due to the psychological and emotional status caused by the Coronavirus pandemic [27]. Nonetheless, in the Emodi-Perlman et al. study [27], despite using a large sample size, the used screening questionnaire (the PHQ-4), with just four questions for the assessment of anxiety and depression, may not allow for a deep evaluation of psychological factors. Altogether, the COVID-19 pandemic has generated a stress context common to the entire population, thus constituting a naturally suitable context for the study of the relationship between psychological factors and bruxism.

Therefore, the aim of this study was to investigate the impact of the COVID-19 pandemic on three samples of first-year dental students pre-, mid-, and post-pandemic, both on their possible sleep and awake bruxism and the associated psychological factors assessed with comprehensive valid, and reliable questionnaires. To this aim, first-year dentistry students from the Faculty of Dentistry of the Complutense University of Madrid in the 2018/19 (pre-pandemic) and 2020/21 (pandemic), and 2021/22 (post-pandemic) academic years were thoroughly assessed for anxiety, depression, personality, stress coping, as well as awake and sleep bruxism. (It should be noted that most restrictions were gradually lifted in Spain during 2021 summer (except for the use of sanitary masks in public transportation still persisting) and the non-online presential dentistry academic year started at the beginning of September 2021. Even though the COVID-19 pandemic might be still mildly active regardless of the restriction lift, for simplicity, we decided to call the 2021/22 group of students, post-pandemic group instead of post-pandemic group). 

Considering previous results, higher levels of possible awake and sleep bruxism were expected for the pandemic group (2020/21) concerning the pre-pandemic (2018/19) and post-pandemic groups (2021/22). Furthermore, increased levels of psychological variables, such as anxiety and depression, were expected for both the pandemic group and the post-pandemic group in comparison to the pre-pandemic group and bruxers compared to non-bruxers. Furthermore, a lack of differences between pandemic and post-pandemic groups might indicate that pandemic psychological effects still persist.

## 2. Materials and Methods

### 2.1. Study Design

This was an observational case-control study to investigate the impact of COVID-19 on bruxism activity in first-year dental students, along with the assessment of various psychological symptoms related to it. The study was carried out at the Faculty of Dentistry of the Complutense University of Madrid, with the approval of the ethics committee of the “CEIC San Carlos Clinic Hospital” (Ref. C.I. 15-159-E). 

### 2.2. Participants

First-year student volunteers were recruited from the years 2018/19 (pre-pandemic group), 2020/21 (group affected by the COVID-19 pandemic), and 2021/22 (post-pandemic group). Exclusion criteria for all groups were: alcohol abuse or drug use, pregnancy, medical treatment with antidepressant, anxiolytic, opiate medications, and a history of severe psychiatric illnesses. A total sample of 274 students participated in the experiment; 92 students (out of 100 enrolled this year) from the pre-pandemic 2018/19 academic year (72 women, 20 men), between 17 and 31 years old ($\bar{X}$ = 19.44, σ = 2.5); 91 students (out of 100 enrolled) from the pandemic 2020/21 academic year (71 women, 20 men), between 17 and 27 years old ($\bar{X}$ = 18.71, a σ = 1.52 years); and 91 students (out of 100 enrolled) from the post-pandemic 2021/22 academic year, between 17 and 33 years old ($\bar{X}$ = 19.03, σ = 2.7). No significant differences were observed among groups for age (χ^2^ = 2.8, *p* = 0.24) and percentage of males and females (χ^2^ = 0.37, *p* = 0.83), between pre-pandemic, pandemic, and post-pandemic groups.

### 2.3. Materials

#### 2.3.1. Self-Reported Bruxism Questionnaire

To evaluate possible bruxism, first the Pintado et al. [28] questionnaire was used. It contains six items evaluating sleep (items 1 to 4) and awake bruxism (items 5 and 6). Second, the jaw muscle tension questionnaire [12] was used to assess the sensations of tension or stiffness in the jaw muscles. It includes the following questions: (1) How would you rate your jaw muscle stiffness or tension at the present time? (2) What was the greatest jaw muscle tension or stiffness felt in the last 6 months? (3) What was the average jaw muscle intensity or stiffness felt during the last 6 months? The questions included a visual analog scale, ranging from 0 to 10 points, where 0 would indicate the “absence of tension” and 10 would mean “the highest possible tension.” Participants were classified as probable awake bruxers when they answered “Yes” to items 5 or 6 in the Pintado questionnaire, both of which refer to the awareness of clenching or grinding teeth during wakefulness and showed a score equal to or larger than 4 regarding the average intensity of the tension and stiffness experienced in the last 6 months (third question), since, in a previous study, participants selected with this criteria seemed to present larger masseter electromyographic activity than controls [19], which would enhance diagnosis certainty. Possible sleep bruxers were evaluated based on items 1–4 of Pintado questionnaire, which refer to sleep bruxism.

Given the pandemic restrictions, only self-reported bruxism was assessed by means of questionnaires, therefore the participants’ diagnoses were classified as possible bruxism, although for simplicity, sleep and awake bruxism terms are mainly used in the remainder of this study.

#### 2.3.2. Psychological Questionnaires

The selected questionnaire to evaluate anxiety and its symptoms, depression and its symptoms, somatization symptoms, stress coping, and personality have been validated for Spanish (Spain) samples and have high levels of reliability and validity in all their scales (>0.8), being largely used in research [10,11,12,29,30,31,32,33,34].

The STAI questionnaire is composed of 10 items assessing state anxiety STAI-E (transient emotional state) and another 10 items for trait anxiety STAI-R (anxious, relatively stable propensity of the participant in general) [30,35]. The State/Trait Depression Questionnaire (ST-DEP) was used to assess depression [31,36]. This 20-item questionnaire has a construction similar to that of the STAI, includes depression scales for state and trait depression, and within each scale includes two euthymia (absence of positive affect) and dysthymia (presence of negative affect) subscales. Additionally, to further evaluate depression, anxiety, and somatization symptoms, the Brief Symptom Inventory 18 (BSI-18) [32,37], a brief version of the well-known SCL-90 [38], was also applied. The Coping Responses Inventory—Adults (CRI-A) [33,39] was used to assess stress coping styles. This questionnaire contains 48 items, and it allows the evaluation of eigt different coping strategies that can be grouped into cognitive/behavioral coping (logical analysis, positive reappraisal, seeking guidance and support, and problem-solving) and approach/avoidance coping (cognitive avoidance, acceptance, or resignation, seeking alternative rewards, and emotional discharge). Lastly, personality was evaluated using the well-known NEO-FFI questionnaire [34,40], which includes 60 items and evaluates the five major personality factors that have shown greater consistency in systematic research: neuroticism, extraversion, openness to experience, agreeableness, and conscientiousness.

### 2.4. Procedure

Participants of the pre-pandemic batch, after receiving instructions, filled out the questionnaires at the same time in a quiet environment. Although no time limit was set, it took participants around 80 min to complete all the questionnaires. The questionnaires were scheduled so that they were administered outside of university exam periods, which might increase stress levels.

Pandemic and post-pandemic groups filled out the questionnaires at their home, after receiving the proper instructions through videoconference. They were asked to fill out the questionnaires in a quiet environment during the next 80 min, while the experimenter remained connected to solve possible doubts or concerns and to submit it within the next 48 h.

### 2.5. Statistical Analysis

Since a multivariate study was conducted, the sample size was calculated with the method described in Naing, et al. [41], conservatively assuming an awake bruxism prevalence of 11% in the general population [2], a level of confidence of 0.95 and a precision of 0.1. Sample size calculation resulted in 38 subjects per group.

The statistical analyses were calculated using SPSS 26 software (SPSS Inc., Chicago, IL, USA) in its versions for Mac Os. Since, according to the Mardia test, normality cannot be assumed (kurtosis, z = 4729.2, *p* < 0.01; and skewness z = 18.1, *p* < 0.01), MANOVA analyses with the inter-factors Group and Bruxism could not be calculated. Therefore, to compare questionnaires’ direct scores of pre-pandemic (2018/19), pandemic (2020/21), and post-pandemic (2021/22) groups, data were analyzed with the Kruskal–Wallis χ^2^ test. The same data analyses were used also to compare age, sex, and the awake and sleep bruxism prevalence among, and the response to the three questions about mandibular tension or stiffness (see detailed description above) [12]. For further comparison of groups two by two, Mann–Whitney and Wilkinson’s tests were calculated when required.

## 3. Results

### 3.1. Awake and Sleep Bruxism Prevalence

**Figure 1:**
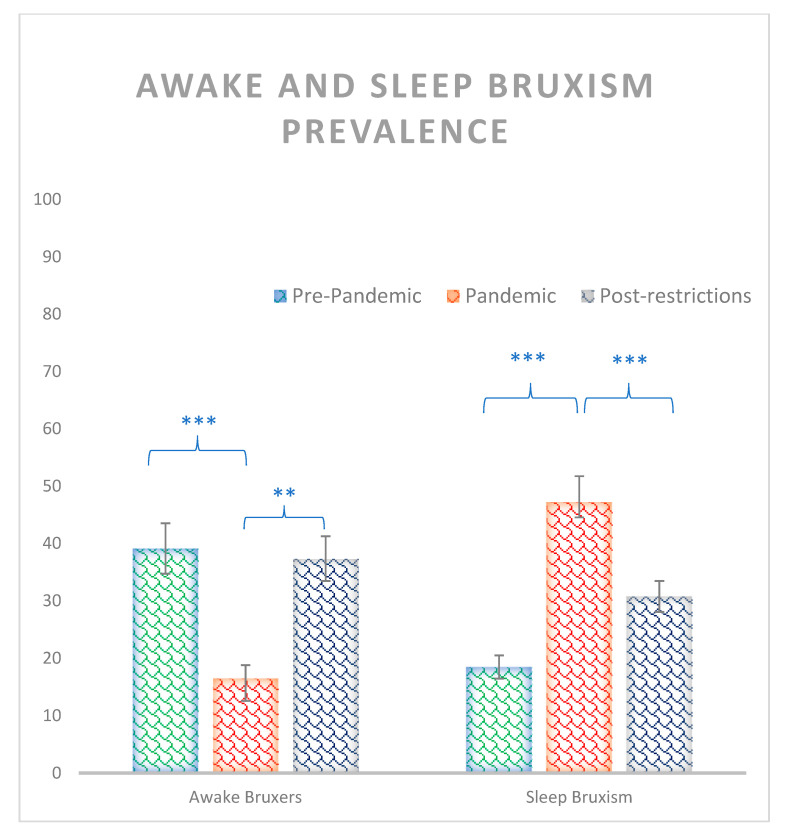
Percentage of awake and sleep bruxism prevalence for pre-pandemic, pandemic, and post restrictions groups. Where *** = *p* < 0.001, ** = *p* < 0.01. Bars indicate error rates.

Significant effects were observed between groups for both sleep and awake bruxism prevalence (χ^2^ = 13.48, *p* = 0.001, and χ^2^ = 13.48, *p* < 0.001 respectively) where the pandemic group (16.5%) showed a smaller percentage of awake bruxers than both pre-pandemic (39.1%) and post-pandemic groups (37.4%) (z = −3.3, *p* = 0.001, and z = −32, *p* = 0.002 respectively), while sleep bruxism was larger for the pandemic group (47.2%) than pre-pandemic (18.4%) and post-pandemic group (30.7%) (z = −4.2, *p* < 0.001, and z = −2.23, *p* = 0.02, respectively). No other significances were observed for awake and sleep bruxism between pre-pandemic and post-pandemic groups (all zs < 0.9, all ps > 0.4) (Figure 1).

### 3.2. Questionnaires

**Figure 2:**
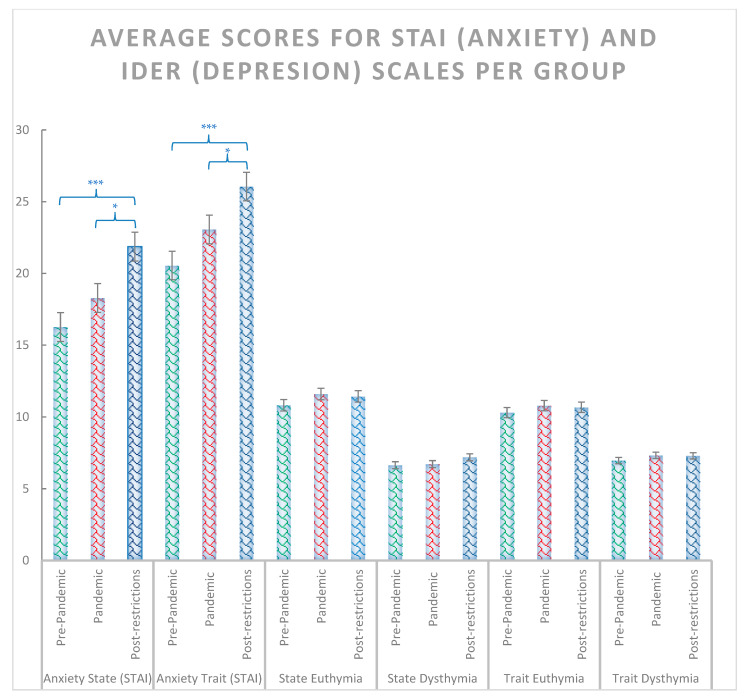
Average scores for STAI (anxiety) and IDER scales (depression) for pre-pandemic, pandemic, and post-pandemic group. Where *** = *p* < 0.001, and * = *p* < 0.05. Bars indicate error rates.

**Figure 3:**
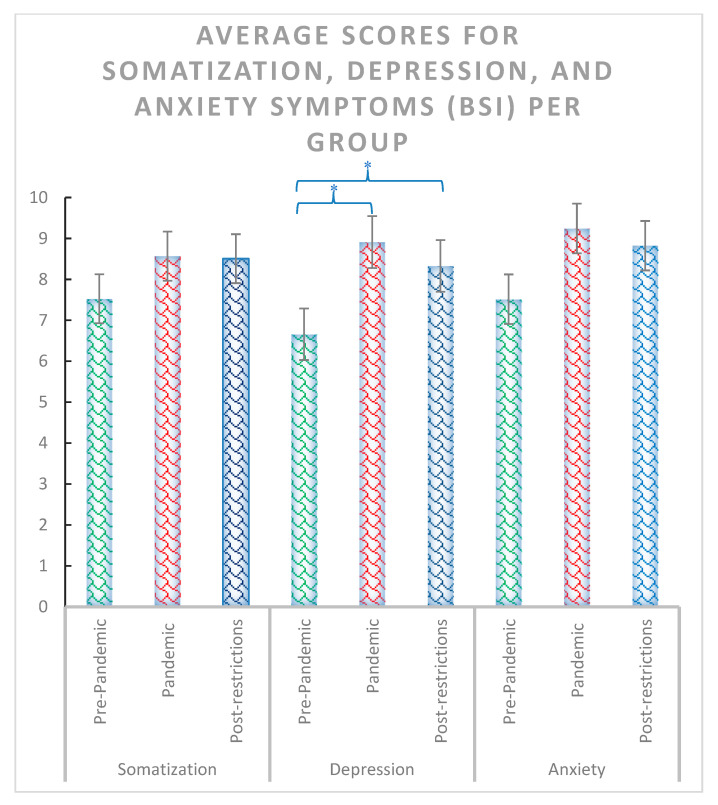
Average scores for somatization, depression, and anxiety symptoms assessed with the BSI-18 questionnaire for pre-pandemic, pandemic, and post-pandemic groups. Where * = *p* < 0.05. Bars indicate error rates.

**Figure 4:**
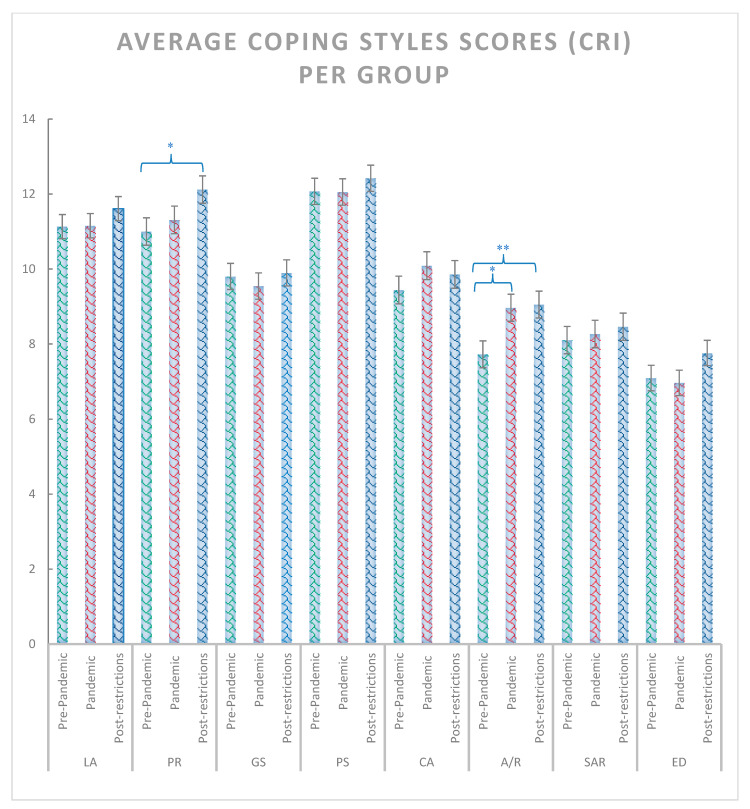
Average scores for logical analysis (LA), positive reappraisal (PR), seeking guidance and support (GS), problem solving (PS), acceptance/resignation (AR), seeking for alternative reward (SAR) and emotional discharge (ED) coping scales assessed with the CRI stress coping questionnaire for pre-pandemic, pandemic, and post-pandemic groups. Where ** = *p* < 0.01, and * = *p* < 0.05. Bars indicate error rates.

**Figure 5:**
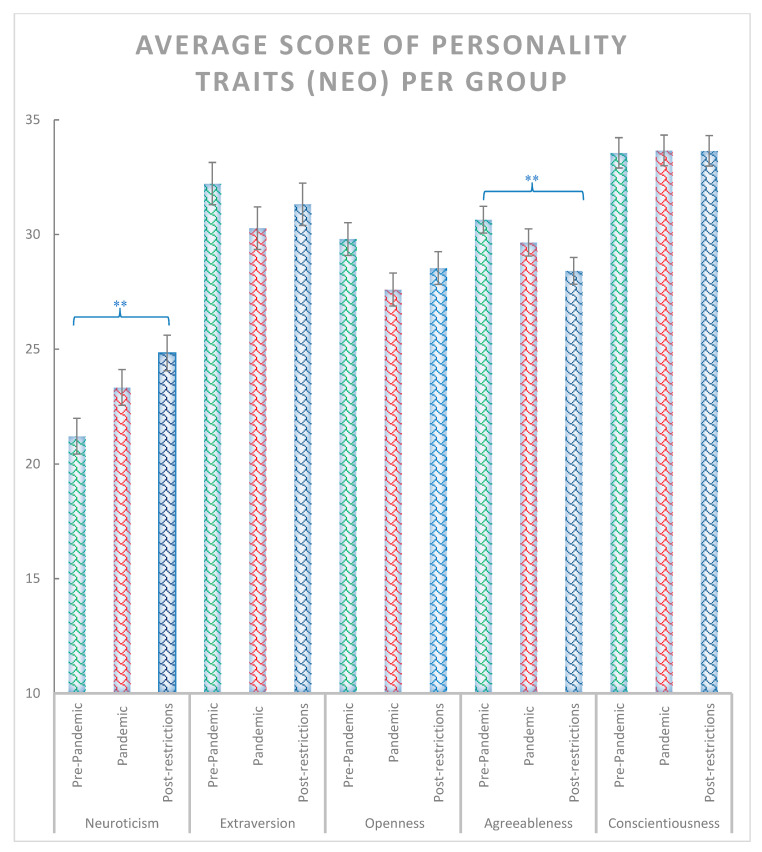
Average scores for neuroticism, extraversion, openness, agreeableness, conscientiousness personality traits assessed with the NEO personality questionnaire for pre-pandemic, pandemic, and post-restrictions group. Where ** = *p* < 0.01. Bars indicate error rates.

The χ^2^ test including the pre-pandemic, pandemic and post-pandemic groups revealed significant effects for anxiety state and trait (χ^2^ = 14.44, *p* < 0.001 and χ^2^ = 13.88, *p* < 0.001, respectively) (Figure 2), depression symptoms (χ^2^ = 7.18, *p* = 0.028) (Figure 3), positive reappraisal (χ^2^ = 6.42, *p* = 0.04), acceptance and resignation (χ^2^ = 7.72, *p* = 0.011) (Figure 4), neuroticism (χ^2^ = 9.72, *p* = 0.008), and agreeableness (χ^2^ = 6.42, *p* = 0.04) (Figure 5). Further detailed analyses found that the post-pandemic group showed larger anxiety state and trait (STAI) than pandemic (state: z = −2.37, *p* = 0.018 and trait: z = −2.061, *p* = 0.039) and pre-pandemic groups (state: z = −3.861, *p* < 0.001 and trait: z = −3.600, *p* > 0.001). Furthermore, higher scores were observed for the post-pandemic group compared to the pre-pandemic group for neuroticism (z = −3.105, *p* = 0.002), agreeableness (z = −2.44, *p* = 0.015) and positive reappraisal coping style, z = −2.551, *p* = 0.011). Finally, students from the pre-pandemic group presented lower levels of depression and acceptation/resignation comping style (A/R) than the pandemic (depression: z = −2.404, *p* = 0.016 and A/R: z = −2.524, *p* = 0.012) and post-pandemic groups (depression: z = −2.207, *p* = 0.027 and A/R: z = −2.658, *p* = 0.008, respectively). For non-significant results and further details of the analyses of psychological variables between groups of students, see Table 1.

The Mann–Whitney analyses comparing awake bruxers vs. non-bruxers did not observe significant effects for any psychological scale (all zs > |1.2|, all ps > 0.247). Similarly, no significances were observed between sleep bruxers and non-bruxers for any psychological variable (all zs > |1.25|, all ps > 0.2) except for a nearly significant result in agreeability (z = 1.96, *p* = 0.05).

## 4. Discussion

The pandemic group showed smaller possible awake bruxism and larger possible sleep bruxism prevalence than both pre-pandemic and post-pandemic. Among the three groups of students, the post-pandemic group was the one that showed higher scores in the psychological variables. Thus, the post-pandemic group of students showed higher levels of state and trait anxiety compared to both pandemic and pre-pandemic groups. They also presented higher levels of neuroticism and agreeableness personality traits, and positive reappraisal than the pre-pandemic group, being the pandemic group somewhere in between since differences in these scales were not observed either when compared to the pre-pandemic, or the pos-restriction group. Additionally, students from the pandemic group and post-pandemic groups showed higher levels of depression and acceptance/resignation comping style than the pre-pandemic one.

Although for the post-pandemic group, most of the pandemic restrictions were lifted gradually during the previous month, and at the data collection moment no restriction remained except for the use of hygienic masks in public transport, the psychological consequences of the pandemic persisted and they were similar (depression, acceptation/resignation) or even worse (trait and state anxiety, agreeableness, and neuroticism) than in the pandemic group. These findings are in line with previous studies where increased levels of anxiety, stress, and depression were observed in the population as a consequence of the COVID-19 pandemic [22,23,24,25,42,43,44,45]. Previous studies also found that neuroticism is associated with higher perceived stress and emotional instability during the pandemic [46,47], accordingly larger levels of neuroticism and agreeableness were observed for the post-pandemic group. A neuroticism personality is characterized by emotional instability, including the tendency for anxiety and excessive preoccupation over daily situations, while agreeableness is the individual’s tendency to develop and maintain prosocial relationships [34]. The pandemic situations might have enhanced the levels of preoccupation and emotional instability while reducing prosocial relationships. Personality traits are characterized by both stability and change across the lifespan, where levels seem to be quite stable in adulthood, but with age, they tend to decrease for neuroticism, while they tend to increase for agreeableness (for USA samples) [48]. Nonetheless, for the post-pandemic group, the statistical mode was 18 years old, thus at the beginning of the pandemic, they were around 16 years old, where personality is still developing [49]. Therefore, our data might indicate that the COVID-19 pandemic might have affected personality in teenagers or at least limited age-related personality changes. The present findings cannot disentangle whether the effects on psychological variables for the post-pandemic group compared to the pre-pandemic and pandemic group could be due to a larger exposition to pandemic consequences and restrictions, or/and to a larger vulnerability due to their younger age at the pandemic onset.

Even though the psychological variables of the post-pandemic group seem to be more affected by the pandemic, some signs of recovery are also observed, since positive reappraisal was higher. Positive reappraisal is a strategy used to cope with negative events by attempting to see a problem in a positive way while still accepting the reality of the situation [33]. This strategy is generally considered an adaptive cognitive strategy in stress-coping models [50], which might constitute an initial sign of recovery.

The pre-pandemic group had a prevalence of awake and sleep bruxism, similar to those reported in other studies in adolescents and university students [51,52,53,54]. Although the prevalence of sleep bruxism was larger for the post-pandemic group than for the pre-pandemic group, significant differences were not observed; therefore, according to the present data, both groups behaved similarly. Remarkably, the pandemic group significantly presented fewer awake bruxers but more sleep bruxers than pre-pandemic and post-pandemic groups. In contrast, a recent study found that both awake and sleep bruxism were more prevalent in female patients during the COVID-19 pandemic, which was concomitant with higher levels of anxiety [26]. On the one hand, our criteria for awake bruxism were stricter than in the former study since students were classified as probable awake bruxers when they answered “Yes” to items 5 or 6 in the Pintado questionnaire for awake bruxism and showed a score equal or higher than 4 in the third question of the mandibular tension questionnaire (average tension/stiffness experienced in the last 6 months). Those stricter criteria were chosen because previous studies observed larger masseter electromyographic activity in bruxers selected with this method [19]. It is possible that the COVID-19 pandemic promoted milder awake bruxism in the pandemic group, which might have been ruled out by our restrictive criteria, but the same criteria were used for the other two groups. Additionally, the pandemic sample was formed by students with no, or few, daily activities outside their homes, either because of the lockdown, quarantine, or the non-presential online classes. Awake bruxism seems to be triggered in experimental situations by active stressful activities [18]. Furthermore, the pandemic group presented larger depression than the pre-pandemic group, but significant differences in anxiety were not observed. It is possible that “stressful situations” resulting from an active life increasing anxiety might promote more awake bruxism, while stress due to resignation and lack of activity, depressive mood, or passive coping might promote sleep bruxism. In this line, the pandemic sample presented a larger bruxism prevalence and showed higher levels of acceptance/resignation coping style and depression which has been related to a passive coping style, while the post-pandemic sample, allowed to have a more active lifestyle, started to present more active/adaptive coping style (larger positive reappraisal). Passive coping seems to be more related (but not only) to a predominant activation of the hypothalamic-pituitary-adrenocortical (HPA) axis in charge of the release of glucocorticoid (cortisol), while the sympathetic adrenomedullary stress system (SAM) is responsible for adrenaline and noradrenaline release and has been more related to active/adaptative coping [55,56,57,58]. In this line, healthy awake bruxers with high anxiety levels showed increased active/adaptive coping styles than controls [12]. Furthermore, although the data are still controversial, increases in adrenaline and noradrenaline levels have been related to both awake and sleep bruxism [59] but enhanced cortisol levels are generally associated with sleep bruxism [11]. However, further research is needed to confirm this possible relationship between active coping and awake bruxism and passive coping and sleep bruxism.

Except for a nearly significant effect on agreeableness in sleep bruxers, we failed to find significant differences between bruxers and non-bruxers (awake and sleep bruxers) in psychological variables. However, there is a broad consensus regarding the important role played by stress, anxiety, or depression in the development of bruxism [5,11,12,13,14]. Indeed, using a similar sample and methodology, higher levels of anxiety, depression, and neuroticism, as well as positive reappraisal in awake bruxers were previously observed by our research team [12]. The lack of significance could be explained because two thirds of the sample was under the pandemic effects, which might have caused a ceiling effect by generally increasing stress levels of, anxiety, and depression, thus masking possible differences between bruxers and non-bruxers.

### Limitations, Strengths and Future Directions

The psychological assessment was very thorough, including many valid questionnaires which, together with the pandemic circumstances, hindered data collection. However, larger sample sizes might improve the normality of the data, thus allowing for more powerful statistical analyses and a comparison of psychological factors for bruxers and non-bruxers within each group. Due to the pandemic situation, only possible bruxism could be assessed. Additionally, although it was out of the scope of our study, other possible behavioral factors related to the bruxism habit should be also controlled in future studies. For instance, the use of tooth wear (for different findings see references [60,61]) and changes in lifestyle observed during the COVID-19 pandemic, such as changes in sleep habits, use of electronic devices, caffeine, and other drugs intake [62]. Although the sample selection (a cohort of university students) favoured the homogeneity of the samples in terms of age, sociological, cultural, and environmental variables, further research including larger and more representative samples of participants, including a similar number of males and females, could enhance the generalizability of the results.

## 5. Conclusions

Regardless of the limitations, it can be concluded that the COVID-19 pandemic affected sleep and awake bruxers. The higher prevalence of sleep bruxism in the pandemic group might be related to stressful situations passively suffered, promoting increased levels of depression and an acceptance/resignation coping style, which is normally considered a passive coping style (often seen as maladaptive), while stressful situations derived from a more active life might promote increased anxiety and the larger levels of awake bruxism observed in both pre-pandemic and post-pandemic groups. However, further research is needed to confirm this possible relationship. Regarding the psychological variables, the more affected group was the post-pandemic one, which presented higher levels of anxiety (state and trait), depression, acceptation/resignation coping style, higher neuroticism (emotional instability trait), and lower agreeableness traits than the pre-pandemic group, while the pandemic group was somehow in between. This could be a consequence of the longer exposition to the pandemic consequences or an increased vulnerability due to the younger age at the onset of the pandemic, at a time when personality is still developing. Nonetheless, the post-pandemic group presented higher levels of positive reappraisal, an active adaptive coping strategy that might be an initial sign of recovery.

## Figures and Tables

**Table 1:** Statistical results of the psychological scales. Data were analyzed by the Kruskal–Wallis χ^2^ test. For further two-by-two comparisons, Mann–Whitney tests were calculated.

Scale (Questionnaire) Group		Average	Std	Square-Chi	*p*	Post-Hoc	z	*p*
Anxiety State (STAI)	Pre-Pandemic (1)	16.26	7.971	14.447	0.001	(1) vs. (2)	−1.064	0.287
	Pandemic (2)	18.29	10.314			(2) vs. (3)	−2.375	0.018
	Post-restrictions (3)	21.87	10.254			(1) vs. (3)	−3.861	0.000
	Total	18.81	9.816					
Anxiety Trait (STAI)	Pre-Pandemic (1)	20.55	9.084	13.88	0.001	(1) vs. (2)	−1.898	0.058
	Pandemic (2)	23.07	9.102			(2) vs. (3)	−2.061	0.039
	Post-restrictions (3)	26.05	10.248			(1) vs. (3)	−3.600	0.000
	Total	23.22	9.726					
State Euthymia (ST/DEP)	Pre-Pandemic (1)	10.82	3.147	2.26	0.323	(1) vs. (2)	−1.476	0.140
	Pandemic (2)	11.61	3.908			(2) vs. (3)	−0.439	0.661
	Post-restrictions (3)	11.44	4.177			(1) vs. (3)	−1.006	0.315
	Total	11.29	3.770					
State Dysthymia (ST/DEP)	Pre-Pandemic (1)	6.65	1.997	3.162	0.206	(1) vs. (2)	−0.241	0.810
	Pandemic (2)	6.72	2.422			(2) vs. (3)	−1.574	0.115
	Post-restrictions (3)	7.20	2.544			(1) vs. (3)	−1.481	0.139
	Total	6.86	2.336					
Trait Euthymia (ST/DEP)	Pre-Pandemic (1)	10.31	3.316	2.153	0.341	(1) vs. (2)	−1.571	0.116
	Pandemic (2)	10.80	3.209			(2) vs. (3)	−0.400	0.689
	Post-restrictions (3)	10.68	3.687			(1) vs. (3)	−0.805	0.421
	Total	10.60	3.405					
Trait Dysthymia (ST/DEP)	Pre-Pandemic (1)	6.97	1.816	1.298	0.523	(1) vs. (2)	−0.980	0.327
	Pandemic (2)	7.33	2.293			(2) vs. (3)	−0.074	0.941
	Post-restrictions (3)	7.30	2.111			(1) vs. (3)	−0.989	0.323
	Total	7.20	2.081					
Somatization (BSI)	Pre-Pandemic (1)	7.53	4.586	1.689	0.43	(1) vs. (2)	−0.541	0.588
	Pandemic (2)	8.57	7.196			(2) vs. (3)	−0.615	0.539
	Post-restrictions (3)	8.51	4.981			(1) vs. (3)	−1.368	0.171
	Total	8.20	5.697					
Depression (BSI)	Pre-Pandemic (1)	6.66	4.890	7.128	0.028	(1) vs. (2)	−2.404	0.016
	Pandemic (2)	8.91	7.466			(2) vs. (3)	−0.128	0.898
	Post-restrictions (3)	8.33	5.398			(1) vs. (3)	−2.207	0.027
	Total	7.96	6.070					
Anxiety (BSI)	Pre-Pandemic (1)	7.52	4.254	4.263	0.119	(1) vs. (2)	−1.161	0.246
	Pandemic (2)	9.24	7.967			(2) vs. (3)	−0.714	0.475
	Post-restrictions (3)	8.82	4.301			(1) vs. (3)	−2.131	0.083
	Total	8.53	5.792					
Logical Analysis (CRI)	Pre-Pandemic (1)	11.131	2.8586	1.701	0.427	(1) vs. (2)	−0.051	0.959
	Pandemic (2)	11.156	3.1903			(2) vs. (3)	−1.067	0.286
	Post-restrictions (3)	11.612	3.1464			(1) vs. (3)	−1.185	0.236
	Total	11.300	3.0648					
Positive Reappraisal (CRI)	Pre-Pandemic (1)	11.000	2.8206	6.421	0.04	(1) vs. (2)	−0.831	0.406
	Pandemic (2)	11.311	3.8443			(2) vs. (3)	−1.559	0.119
	Post-restrictions (3)	12.119	3.7183			(1) vs. (3)	−2.551	0.011
	Total	11.477	3.5087					
Seeking Guidance (CRI)	Pre-Pandemic (1)	9.802	3.3241	0.363	0.834	(1) vs. (2)	−0.517	0.605
	Pandemic (2)	9.544	3.4516			(2) vs. (3)	−0.277	0.782
	Post-restrictions (3)	9.897	3.2118			(1) vs. (3)	−0.464	0.642
	Total	9.749	3.3212					
Problem Solving (CRI)	Pre-Pandemic (1)	12.071	3.0492	1.238	0.538	(1) vs. (2)	−0.143	0.886
	Pandemic (2)	12.056	3.1493			(2) vs. (3)	−1.002	0.317
	Post-restrictions (3)	12.421	3.7580			(1) vs. (3)	−0.913	0.361
	Total	12.183	3.3262					
Cognitive Avoidance (CRI)	Pre-Pandemic (1)	9.440	3.2803	1.61	0.447	(1) vs. (2)	−1.248	0.212
	Pandemic (2)	10.089	3.7286			(2) vs. (3)	−0.244	0.807
	Post-restrictions (3)	9.857	3.5373			(1) vs. (3)	−0.892	0.372
	Total	9.794	3.5167					
Acceptance/Resignation (CRI)	Pre-Pandemic (1)	7.725	3.2492	9.028	0.011	(1) vs. (2)	−2.524	0.012
	Pandemic (2)	8.967	3.4882			(2) vs. (3)	−0.264	0.792
	Post-restrictions (3)	9.052	3.5152			(1) vs. (3)	−2.658	0.008
	Total	8.580	3.4606					
Seeking Alternative Reward (CRI)	Pre-Pandemic (1)	8.104	3.5391	0.282	0.869	(1) vs. (2)	−0.308	0.758
	Pandemic (2)	8.267	3.4505			(2) vs. (3)	−0.270	0.787
	Post-restrictions (3)	8.459	3.4475			(1) vs. (3)	−0.505	0.613
	Total	8.277	3.4696					
Emotional Discharge (CRI)	Pre-Pandemic (1)	7.093	3.1893	3.275	0.194	(1) vs. (2)	−0.184	0.854
	Pandemic (2)	6.967	3.3569			(2) vs. (3)	−1.621	0.105
	Post-restrictions (3)	7.759	3.1255			(1) vs. (3)	−1.500	0.134
	Total	7.274	3.2318					
Neuroticism (NEO)	Pre-Pandemic (1)	21.21	7.894	9.722	0.008	(1) vs. (2)	−1.639	0.101
	Pandemic (2)	23.33	7.635			(2) vs. (3)	−1.490	0.136
	Post-restrictions (3)	24.84	6.534			(1) vs. (3)	−3.105	0.002
	Total	23.13	7.500					
Extraversion (NEO)	Pre-Pandemic (1)	32.22	10.718	1.853	0.396	(1) vs. (2)	−1.232	0.218
	Pandemic (2)	30.28	8.148			(2) vs. (3)	−0.442	0.658
	Post-restrictions (3)	31.32	7.043			(1) vs. (3)	−1.038	0.299
	Total	31.28	8.778					
Openness (NEO)	Pre-Pandemic (1)	29.80	7.140	3.546	0.17	(1) vs. (2)	−1.808	0.071
	Pandemic (2)	27.60	6.775			(2) vs. (3)	−0.865	0.387
	Post-restrictions (3)	28.54	6.588			(1) vs. (3)	−1.140	0.254
	Total	28.65	6.873					
Agreeableness (NEO)	Pre-Pandemic (1)	30.65	5.890	6.42	0.04	(1) vs. (2)	−1.415	0.157
	Pandemic (2)	29.66	4.968			(2) vs. (3)	−1.292	0.196
	Post-restrictions (3)	28.42	5.879			(1) vs. (3)	−2.441	0.015
	Total	29.57	5.652					
Conscientiousness (NEO)	Pre-Pandemic (1)	33.56	6.620	0.007	0.996	(1) vs. (2)	−0.020	0.984
	Pandemic (2)	33.67	6.316			(2) vs. (3)	−0.121	0.904
	Post-restrictions (3)	33.65	6.043			(1) vs. (3)	−0.006	0.996
	Total	33.63	6.308					

## Data Availability

The data presented in this study are available on request from the corresponding authors.
